# Widespread sensorimotor and frontal cortical atrophy in Amyotrophic Lateral Sclerosis

**DOI:** 10.1186/1471-2377-6-17

**Published:** 2006-04-25

**Authors:** Julian Grosskreutz, Jörn Kaufmann, Julia Frädrich, Reinhard Dengler, Hans-Jochen Heinze, Thomas Peschel

**Affiliations:** 1 Medical School Hannover, Dept. of Neurology, Hannover, Germany; 2 Otto-von-Guericke University, Dept. of Neurology II, Magdeburg, Germany; 3 Center for Systems Neuroscience (ZSN), Hannover, Germany; 4 Medical School Hannover, Dept. of Psychiatry and Psychotherapy, Hannover, Germany

## Abstract

**Background:**

Widespread cortical atrophy in Amyotrophic Lateral Sclerosis (ALS) has been described in neuropathological studies. The presence of cortical atrophy in conventional and scientific neuroimaging has been a matter of debate. In studies using computertomography, positron emission tomography, proton magnetic resonance spectroscopy and conventional T2-weighted and proton-weighted images, results have been variable. Recent morphometric studies by magnetic resonance imaging have produced conflicting results regarding the extent of grey and white matter involvement in ALS patients.

**Methods:**

The authors used optimized voxel-based morphometry as an unbiased whole brain approach to detect differences between regional grey and white matter volumes. Seventeen patients with a diagnosis of ALS according to El-Escorial criteria and seventeen age-matched controls received a high resolution anatomical T1 scan.

**Results:**

In ALS patients regional grey matter volume (GMV) reductions were found in the pre- and postcentral gyrus bilaterally which extended to premotor, parietal and frontal regions bilaterally compared with controls (p < 0.05, corrected for the entire volume). The revised ALS functional rating scale showed a positive correlation with GMV reduction of the right medial frontal gyrus corresponding to the dorsolateral prefrontal cortex. No significant differences were found for white matter volumes or when grey and white matter density images were investigated. There were no further correlations with clinical variables found.

**Conclusion:**

In ALS patients, primary sensorimotor cortex atrophy can be regarded as a prominent feature of the disease. Supporting the concept of ALS being a multisytem disorder, our study provides further evidence for extramotor involvement which is widespread. The lack of correlation with common clinical variables probably reflects the fact that heterogeneous disease processes underlie ALS. The discrepancy within all published morphometric studies in ALS so far may be related to differences in patient cohorts and several methodological factors of the data analysis process. Longitudinal studies are required to further clarify the time course and distribution of grey and white matter pathology during the course of ALS.

## Background

Amyotrophic Lateral Sclerosis (ALS) is a fatal neurodegenerative disease which leads to death due to neurogenic muscle weakness within a median time span of three years [1]. Patients clinically develop a paralysis with signs of upper and lower motoneuron loss in limb, trunk and bulbar regions. Oculomotor neurons are spared [2] and neurons of Onuf's nucleus are only slightly affected [3]. Beyond motor deficits, recent reports have described frontal dysfunction to be present in approximately 1/3 of ALS patients [4]. Overlap syndromes exist, such as ALS-Parkinson-Dementia Complex of Guam where an association with a food chain concentration of the neurotoxic amino acid beta-methylamino-l-alanine (BMAA) [5] has been found. Familial cases constitute 5 – 10% of cases which are caused by mutations of the human superoxide dismutase in 20% of familial ALS [6,7]. A recent histopathologic study revealed an overlap of hallmarks of ALS, frontotemporal dementia (FTP) and Alzheimer's disease (AD) in a cohort of patients clinically diagnosed as having ALS [8]. The ALS disease process therefore seems to involve areas beyond the upper motoneuron regions of the cortex and may be the result of heterogenous pathological cellular processes affecting specifically vulnerable neurons both in the spinal cord and the cortex.

Magnetic resonance imaging studies (MRI) in ALS are clinically used to exclude other diseases of the brain. Abnormal signal hyperintensity along the corticospinal tract (CST) in T2-weíghted and proton density (PD) images have been described in ~40% of ALS cases as early as 1991 [9]. Later on, these findings have been seen in ~2/3 of ALS patients [10]. PD images have been found to be slightly more sensitive than T2 images in detecting ALS related CST changes in a study including post-mortem follow-up [4] which described distinct myelin pallor and gliosis as the histological correlate. FLAIR images displayed a hyperintense signal with a higher specificity than other modalities along the descending pyramidal tract of ALS patients [11]. More recently, group comparisons using statistical parametric mapping (SPM) on diffusion tensor images (DTI) have shown changes along the corticospinal tract which correlated well with the clinical distribution of motor deficits in ALS patients [12,13].

In contrast to definite abnormal findings along the CST, studies of cortical atrophy in ALS as detected by MRI have produced conflicting results. On conventional MRI, an abnormally large central sulcus can be often be found as indirect marker of precentral gyrus atrophy [14]. In addition, serial computertomography (CT) and MR imaging showed frontal and limbic system atrophy in ALS [15]. MR spectroscopy revealed abnormal ratios of N-acetylaspartate (NAA) to creatine (Cr) as a marker of neuronal loss in the motor cortex in ALS patients [14,16,17]. Other Imaging modalities such as [11C] flumazenil position emission tomography (PET) [18] displayed a reduced GABA_A _binding in parietal to frontal areas. This finding is supported by histological evidence of reduced expression of the alpha1-subunit of the GABA_A _receptor in ALS motor cortex [19].

Recently, voxel-based analyses of proton density images MRI have demonstrated predominant frontal cortex atrophy in ALS patients but have failed to localize atrophy in the motor cortex [20,21] whereas other authors showed primary motor and extramotor involvement to varying degrees by different methods of voxel based morphometry [22,23].

Voxel based morphometry (VBM) is a fully automated, operator-independent whole brain image analysis technique that allows the voxel-wise comparison of segmented grey and white matter images between two groups of subjects [24-26]. It has the advantage that macroscopic differences are discounted using normalization, and differences in local tissue composition can be explored without resorting to the use of manually placed regions of interest. Furthermore, no a priori hypothesis regarding the localization of group differences is required. VBM has been recently refined and successfully used to study structural brain correlates of aging and changes of grey and white matter volumes in different neurodegenerative diseases [27-29].

The aim of the present study was to identify possible structural brain alterations in a group of mildly affected ALS patients in comparison to normal controls, and to investigate the correlations of anatomical changes with relevant clinical variables by using VBM.

## Methods

### Clinical base

19 patients were recruited from the ALS outpatient clinic of the department of Neurology, Medical School Hannover, where they were classified as having definite, probable, possible or suspected ALS according to the original El Escorial criteria [30]. Suspected ALS was included to allow early disease stage patients to enter the study as epidemiology has suggested a high probability of correct initial diagnosis [31]. In two patients MRI was not performed; one was too heavy for the MRI table and the other did not tolerate lying horizontally due to breathing difficulty. All but one patient progressed to definite or probable ALS according to the revised El Escorial criteria [32] on follow up which is now considered a requirement to include patients into clinical trials. Of the 17 patients in which MRI was done, mean (± S.D.) duration since onset of symptoms, in the following termed disease duration, at MRI was 24 ± 9 months, mean age 61 ± 13 (range 34–77) years and the revised ALS functional rating scale [33] (ALSFRS-R) 40 ± 6 points. The female to male ratio was 3:14; six patients had a bulbar onset and 11 patients limb onset of weakness. Seventeen age matched (p = 0.89, student's t-test) healthy controls (mean age 58 ± 9.4, range 38–68, 7 females) were also studied. Neither patients nor controls had a history of cerebrovascular disease, longstanding hypertension or inflammatory disease of the central nervous system (CNS). All patients were on riluzole; none were taking psychoactive drugs. Written informed consent was obtained from all participants according to the approval of the ethics committee of the Medical School Hannover.

### Clincal status

**Figure 1:**
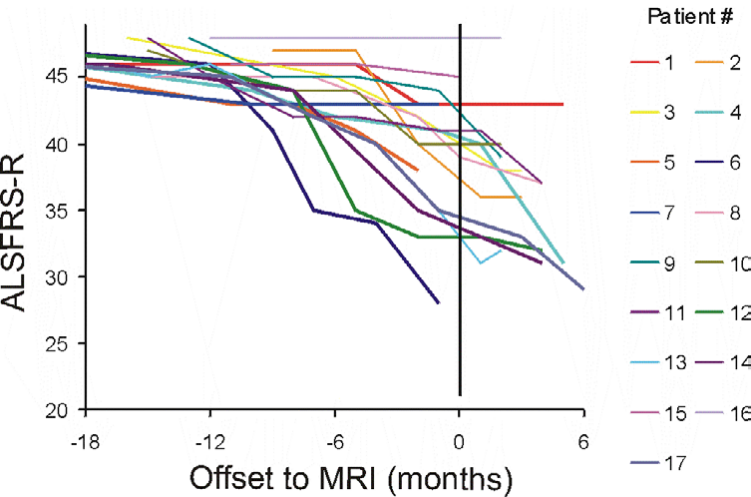
**Course of ALSFRS-R score prior to MRI**. On average, ALSFRS was 39.6 ± 5.6 around the time of the scan reflecting the ALS cohort was mildly affected by disease. Note that some patients had barely gone into rapid progression whereas others have remained stable at a high score.

ALSFRS-R progression rate per month was calculated for the total disease duration [PR/mth(t)] and the six month period prior to MRI [PR/mth(6)]. Sum scores of Medical Research Council (MRC) muscle strength were taken for the upper extremity (shoulder abduction, inward and outward rotation; elbow flexion and extension; lower arm pronation and supination; wrist flexion and extension; finger flexion, extension, abduction and adduction; 5th finger abduction and thumb opposition; best possible score 75 points) and the lower extremity (hip strength; knee flexion and extension; foot flexion, extension, inversion and eversion; toe flexion and extension; best possible score 45 points). Bulbar involvement was described as a sum score of dysarthria (0 = no, 1 = yes), dysphagia (0 = no, 1 = yes), eyelid closure, mouth closure, tongue movement and palate elevation (0 = normal, 1 = reduced, 2 = weak, 3 = absent) with a worst possible score of 14. In a similar fashion, muscle fasciculations (upper arm, lower arm, hand, upper thigh, lower thigh and foot of both sides; rump and tongue; worst score of 42) and muscle atrophy (upper arm, lower arm, hand, upper thigh, lower thigh both sides; worst score of 30) were quantified (0 = normal, 1 = discreet, 2 = moderate, 3 = pronounced). Reflexes were summed by counting the number of muscles scoring 3 or 4 on the NINDS reflex scale for the biceps, triceps, brachialis, finger flexors, quadriceps and gastrocnemius muscles on both sides and masseter muscle (worst score 13). Spasticity was described as normal (0), noticeable (1), pronounced (2), barely to overcome (3) and not to overcome (4) with a worst score of 32 (arm, hand, upper thigh, lower thigh both sides). A positive Babinski sign was counted separately for each side (worst score of 2). The clinical characteristics of the patients are summarized in Table 1, progression of the ALSFRS-R prior to MRI and the clinical status are displayed in Figure 1 and Table 2, respectively.

### Data acquisition

Images were acquired on a neuro-optimized 1.5-T GE Signa Horizon LX (General Electric Company, Milwaukee, WI, USA) using a 3-dimensional T1-weighted spoiled gradient recalled echo (SPGR) sequence generating 124 contiguous sagittal slices (RT 24 ms; TE 8 ms; flip angle 30°, 2 averages, acquisition time 13'10", in plane resolution 0.97 × 0.97 × 1.5 mm^3^). During scanning, all participants were comfortably placed and their heads were fixated within the headcoil with special cushions. All subjects received additional T2-weighted images to exclude cerebrovascular disease, which was normal in all subjects studied according to standard clinical neuroradiological criteria on visual inspection.

### Preprocessing of structural data

Data were processed on a standard IBM-compatible PC using SPM2 statistical parametric mapping software (Welcome Department of Cognitive Neurology, London) and working in an analysis environment (MATLAB; the Math Works Inc, Natick, Mass). The images were reoriented into oblique axial slices aligned parallel to the anterior-posterior commissural axis with the origin set to the anterior commissure. An optimized version [26] of the VBM protocol was followed as recently described by our group [34]. In brief, this involves first the creation of a customized template, followed by an iterative procedure for segmentation and normalization of images. The resulting images were resliced to a final voxel size of 1 mm^3^. Each optimally normalized tissue-specific whole-brain model was then segmented to isolate the corresponding tissue compartment producing grey, white matter, and CSF maps in MNI space. Voxel values in segmented images were multiplied by the Jacobian determinants derived from spatial normalization to provide intensity correction for induced regional volumetric changes, thus preserving within-voxel volumes that may have been altered during non-linear normalization. The analysis of these 'modulated' data tests for regional differences in absolute tissue volume. The images were smoothed to 8 mm using a FWHM (full width half-maximum) Gaussian filter to minimize individual gyral variations and to increase the statistical validity of the analysis. As a consequence of smoothing, each voxel in these 'modulated' images contains an absolute measure of tissue volume from around that voxel.

### Statistical analysis

Processed images of each tissue class were analyzed in the framework of the general linear model. This framework allows the testing, on a voxel-by-voxel basis, of the null hypothesis that the tissue volumes in the two populations (patients and controls) are the same. The resulting statistical parameters constitute a SPM of the t statistic (SPM (t)). Group comparison of ALS patients and healthy controls was performed in SPM2 using the model 'compare-populations: one scan/subject (ANCOVA)'. During modulation we incorporated the correction for volume change induced by spatial normalization. Therefore, it was appropriate to include the global mean voxel value of each tissue class as a covariate to determine the regionally specific pattern of loss or gain within each compartment as well as to remove any variance due to differences in head size. As previous VBM studies in ALS patients used density images [20,22]., we performed additional group comparisons on unmodulated data. Here, we controlled for global differences in voxel intensity across scans by proportional scaling the global mean voxel intensity value.

Additionally, regression analyses with clinical measures were explored using the SPM2 model 'single subject: covariates only'. As for the group comparisons, ANCOVA with the mean voxel value was used to normalize image intensity in the different tissue maps to allow identification of the regional pattern of these correlations. Significance levels were set at P < 0.05 on the voxel level, corrected for multiple comparisons in the entire volume using the false discovery rate method [35].

## Results

Disease duration was taken from onset of symptoms, which preceded the diagnosis of ALS for up to two years. Diagnosis on study inclusion was suspected in some cases, but eventually progressed to probable or definite on follow up in all but one patient. Thus, our patient group is considered mildly affected with an average ALSFRS of ~40 out of a maximum score of 48. On visual inspection of the MR images no subject had focal atrophy of any brain region which may have hindered alignment into standard space or segmentation into grey matter, white matter and CSF.

### Group comparison of ALS patients (n = 17) and controls (n = 17)

**Figure 2:**
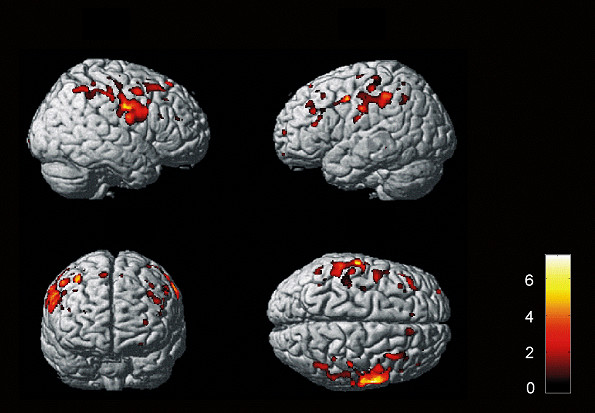
**Regional grey matter atrophy in ALS patients compared to controls**. Group comparison of 17 ALS patients versus 17 healthy controls showed regional grey matter atrophy in the precentral and postcentral gyrus bilaterally, which extended from the primary motor cortex to premotor, parietal and frontal regions bilaterally (displayed at p = 0.001, uncorrected, extended threshold 100 voxels). The color bar represents the T-score. The differences between the groups are superimposed on a standard normalized T1-weighted image. Images are shown in neurological convention.

ALS patients displayed significantly reduced grey matter volumes (GMV) in the precentral gyrus, the postcentral gyrus, parietal and frontal regions bilaterally (Figure 2, Table 3). In the white matter tissue class, no significant voxels were found. In CSF segments, no significant differences were detected.

**Figure 3:**
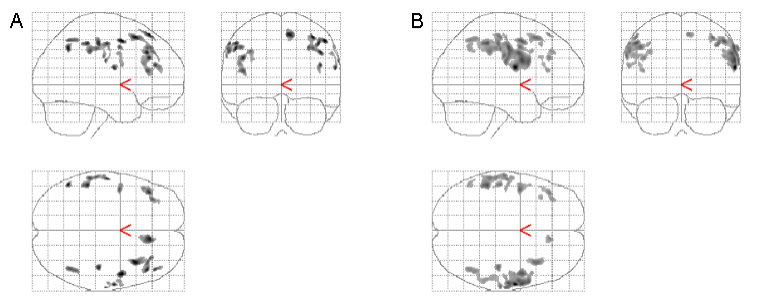
**Group comparison of unmodulated and modulated grey matter segments**. Group differences of grey matter densitiy reductions (unmodulated data, A) and regional grey matter volumes (modulated data, B) in ALS patients versus controls are displayed within a 'glass brain' view. Note that only the comparison of modulated data survived the correction for multiple comparisons specified a priori. Images are displayed at p = 0.001, uncorrected, extended threshold 100 voxels and shown in neurological convention.

Comparable results where found for unmodulated grey matter segments at p < 0.001 uncorrected, but these results did not reach the level of significance specified a priori (Figure 3). No significant differences were found when analysing unmodulated data for white matter and CSF segments.

### Correlation with clinical data

ALS patients clinical data and scores were separately correlated with grey and white matter voxel values. Only the ALSFRS-R as a validated marker of disease progression correlated positively with GMV in the right medial frontal gyrus corresponding to the dorsolateral prefrontal cortex (Brodmann area 10, MNI x = 4, y = 65, z = 20; T = 4.95). Disease duration and onset (bulbar/limb) were not reflected in a specific pattern of atrophy. There was no significant correlation with PR/mth(t), PR/mth(6), strength (tested separately for each extremity, upper and lower extremities and all extremities), lower motoneuron signs (muscle atrophy, fasciculations), upper motoneuron signs (increased reflexes, spasticity, Babinski sign) or bulbar signs.

## Discussion

Voxel-based mapping of structural brain alterations in mildly affected ALS patients revealed statistically significant reductions of grey matter volumes in the primary sensorimotor cortex and frontal lobe bilaterally. There was no correlation between the clinical expression and brain morphology in the patient group except for the ALSFRS-R which correlated with extramotor involvement in the right dorsolateral prefrontal cortex.

### Motor cortex atrophy as a prominent feature of ALS

The most prominent finding of our study was bilateral atrophy of the motor strip which extended to primary sensory areas. Loss of giant pyramidal Betz' cells has been described as the pathologic hallmark of the ALS disease process [36]. Furthermore, it has been shown that the number of cortical neurons in ALS is not reduced [37], and some evidence suggests a change in cell body shape rather than a loss of neurons [38]. These findings represent the end point of structural change, while the present study demonstrates that changes can be detected in vivo even in mildly affected patients, using sensitive imaging methods. In keeping with the increasing interest in the comprehensive assessment of ALS brain anatomy, we used highly automated voxel-based morphometric procedures and examined the entire cerebral parenchyma with a conservative statistical approach. We adopted an improved method for modulating data that allowed direct volume measurements. In our analysis, voxel values specifically expressed regional variations in the absolute amount of brain tissue, in contrast with previous voxel-based studies in which the interpretation of results was not self-evident, as voxel-values expressed variations in structure shape and tissue composition [20-22].

Our results are well in line with a recent VBM study in ALS and frontotemporal lobar degeneration [23]. Using modulated grey matter segments in their analysis, the authors detected marked frontal atrophy and considerable motor cortex atrophy although age-related variance was not adequately represented in the control group. Interestingly, those morphometric imaging studies which failed to show considerable grey matter atrophy showed extensive white matter involvement. Following an optimized protocol within VBM investigating the density (unmodulated data) of grey and white matter segments, Kassubek and colleagues [22] found regional decreased grey matter density in the right-hemispheric primary motor cortex and left medial frontal gyrus in 22 patients with definite ALS. However, while there were few differences in grey matter, extensive changes within white matter particularly along the bilateral CST and in extramotor areas were found in line with neuropathological investigations [39,40] and previous quantitative volumetric MRI studies [20,21,41].

This raises the question as to whether GMV reductions in our study are emphasized at the expense of detecting white matter changes. Although neuropathology can only reveal end-stage changes, white matter involvement is considered to be a significant factor in ALS. Degenerating fibres are not only present in the precentral gyrus and paracentral lobule, but they are also abundant in the postcentral gyrus, and occur in considerable numbers in the adjacent parietal and frontal gyri [39]. As revealed by a recent volumetric MRI study, white matter reductions within motor and non-motor areas are more extensive in cognitively impaired than unimpaired ALS patients compared with control subjects [21]. In the present study, no significant white matter alterations were found. Apart from technical differences, these discrepant findings may be related to our cohort of ALS subjects which was considered to be mildly affected by disease. Furthermore, no overt cognitive abnormalities were present although the patients had no formal neuropsychological testing.

Thus, the discrepancy within all published morphometric studies in ALS so far may be related to differences in the relatively small cohorts of patients, and several methodological factors within the data analysis process. For example, as indicated in Figure 3 we have also analyzed our data using density grey matter segments and have found nearly similar results as with modulated data. However, these results were derived at p < 0.001, uncorrected and did not survive the correction for multiple comparisons on the voxel level specified a priori. This is mandatory in VBM studies because the data used for these analyses are not exactly normally distributed (for details see [25]).

### Extramotor involvement in ALS

There is a growing body of evidence for extramotor involvement ALS by pathological [42,43], neuropsychological [44] and in vivo imaging research using PET [18,45]. Furthermore, in line with our results, alterations of the frontal lobe have been reliably demonstrated by previous quantitative volumetric MRI studies [20,21] where these findings were discussed in detail. As a new finding of our study, there was considerable atrophy of the postcentral gyrus bilaterally. Although the cardinal pathological features of ALS include loss of the giant Betz cells in layer V of the primary motor cortex; the anterior horn cells in the spinal cord and degeneration of the corticospinal tracts, other cortical areas seem to be involved by neurodegeneration to varying degrees. A recent PET study has shown that cortical involvement is not restricted to the primary motor cortex by demonstrating widespread reductions in cortical binding of [11C] flumazenil in ALS patients. Reduced binding of [11C] flumazenil which is an antagonist at the benzodiazepine subunit of the GABA_A _receptor was found in multiple cortical areas including primary and secondary motor areas as well as mesial premotor and parietal cortices [18]. Furthermore, previous PET studies using [18F]2-fluoro-2-deoxyglucose have demonstrated predominant reductions in glucose metabolism as well as reductions in regional cerebral blood flow (rCBF) in the primary sensorimotor cortex and adjacent premotor and parietal areas in patients with classical ALS [46,47]. As sensory pathways in ALS are more or less intact [48,49]., grey matter atrophy of the postcentral gyrus in ALS is likely to reflect changes at the cortical level due to progressive degeneration of the upper motor neuron (UMN) in ALS rather than secondary to peripheral conduction delay. Supporting this assumption, animal studies demonstrated a decrease in the density of inhibitory GABA_A _receptors resulting in an increased excitability in multiple cortical areas which are related with regard to their connections to experimentally injured brain areas in rats [50]. Effects remote from the site of injury are not surprising given that neurons in any damaged region of the cortex have reciprocal synaptic connections with neurons in other brain regions. Such remote effects may potentially be responsible for secondary progressive brain pathology in ALS extending beyond the initially affected neural system [45].

### Correlations with clinical status

We did not find a correlation of brain morphology and relevant clinical variables which were thoroughly quantified (Table 2) in the ALS patients. The lack of correlation with disease duration and lower motor neuron signs is not surprising, given the heterogeneity of the condition and the small sample size, and the lack of correlation with UMN dysfunction probably reflects the limited range scores in the patient sample. In addition, this could be attributed to the small percentage of affected cells and physiologic variation in central gyral anatomy despite adequate smoothing of the data. Only the ALSFRS-R score which largely tests activities of daily living and correlates with both objective measures of disease status and levels of disability [33] displayed a significant positive correlation with regional atrophy in grey matter of the right dorsolateral prefrontal cortex (DLFPC). Although we had no formal neuropsychological testing, the cognitive abnormalities of ALS are now well characterized [44,51]. Frank dementia is rare in ALS and usually frontotemporal in type, but more subtle cognitive deficits frequently relating to executive function can be reliably identified in up to 50% patients and appear relatively early on in the course of the disease [44]. Previous morphometric and functional imaging studies have revealed changes in the DLFPC, which is involved in executive functioning, even in cognitively unimpaired patients [21,52]. Overall, these changes were more marked when cognitively impaired patients with evidence of executive and memory dysfunction were investigated. This led to the assumption that those functional and structural abnormalities in the medial prefrontal cortex which corresponds to this profile of principally executive dysfunction may precede cognitive change [21]. The positive correlation with disease status as expressed by the ALSFRS-R and GMV reduction in the right DLFPC revealed by our study is further supportive of this assumption.

## Conclusion

Assessing regional grey matter volumes in mildly affected ALS patients, sensorimotor cortex atrophy can be regarded as a prominent feature of the disease. Supporting the concept of ALS being a multisytem disorder, our study provides further evidence for extramotor involvement which is widespread. The lack of correlation with common clinical variables probably reflects the fact that ALS underlies a heterogeneous disease process. Longitudinal studies are required to further clarify the time course and distribution of grey and white matter pathology during the course of ALS.

## Competing interests

The author(s) declare that they have no competing interests.

## Authors' contributions

JG and TP drafted the manuscript. JK and TP performed the data acquisition and pre- processing of the data. JG designed and analysed, and JF collected the clinical data set. TP designed the MRI study and performed the statistical analysis. JF participated in the statistical analysis of the data. JG, RD and HJH participated in the design and coordination of the study. RD and HJH were involved in the interpretation of results and general conclusions. All authors read and approved the final manuscript.

## Pre-publication history

The pre-publication history for this paper can be accessed here:



## Figures and Tables

**Table 1:** Demographic information

*patient #*	*age *(*yrs*.)	*sex*	*disease duration *(*mths*.)	*onset*	*ALSFRS-R at MRI*	*clinical diagnosis *(*EL Escorial*)
1	59	m	30	limb	43	possible
2	59	m	13	limb	37	probable
3	31	m	17	limb	42	probable
4	68	m	22	bulbar	40	definite
5	65	m	25	limb	37	probable
6	66	f	41	limb	28	definite
7	53	m	38	limb	43	suspected
8	34	f	18	bulbar	39	probable
9	75	m	14	limb	44	suspected
10	62	m	18	bulbar	40	probable
11	49	m	19	limb	41	suspected
12	73	m	24	bulbar	33	definite
13	60	m	30	limb	33	probable
14	77	m	34	limb	33	probable
15	63	f	12	bulbar	45	probable
16	66	m	23	limb	48	suspected
17	72	m	25	bulbar	48	probable

mean ± S.D.	61 ± 13	f/m: 3/14	24 ± 09	b/l: 6/11	40 ± 06	

Patients with suspected ALS were included in the study. All but one patient (#16) progressed to probable and definite ALS according to the El Escorial criteria on follow-up examination.

S.D. = standard deviation; f = female; m = male; b = bulbar; l = limb

**Table 2:** Clinical scores

#	*RUE *(*75*)	*LUE *(*75*)	*RLE *(*45*)	*LLE *(*45*)	*UE *(*150*)	*LE *(*90*)	*all E*. (*240*)	*fasc*. (*0–42*)	*atr*. (*0–30*)	*bulb*. (*0–14*)	*Bab*. (*0–2*)	*spast*. (*0–32*)	*refl*. (*0–13*)	*ALSFRS-R PR/mth*(*6*)	*ALSFRS-R PR/mth*(*t*)
1	68	69	41	41	137	82	219	5	7	2	0	0	0	0.5	0.2
2	75	64	31	27	139	58	197	7	4	0	0	0	0	1.8	0.8
3	64	62	45	45	126	90	216	15	8	0	2	0	12	1.2	0.4
4	75	73	45	42	148	87	235	10	2	2	2	4	4	0.7	0.4
5	72	75	26	16	147	42	189	15	8	0	1	0	6	0.7	0.4
6	67	59	14	13	126	27	153	4	6	0	0	0	6	1.2	0.5
7	74	75	26	23	149	49	198	12	2	0	0	0	0	0.2	0.1
8	70	71	45	45	141	90	231	1	2	4	2	0	12	1.0	0.5
9	60	60	36	39	120	75	195	17	6	0	0	0	0	1.0	0.3
10	63	73	45	45	136	90	226	6	5	4	0	0	12	0.7	0.4
11	54	55	45	45	109	90	199	4	6	0	2	0	0	0.3	0.4
12	58	43	38	38	101	76	177	11	10	3	2	0	6	0.5	0.6
13	58	57	45	45	115	90	205	8	7	2	2	0	5	1.5	0.5
14	70	71	22	37	141	59	200	7	6	0	2	2	12	1.7	0.4
15	75	75	45	45	150	90	240	1	0	3	0	0	11	0.2	0.3
16	61	71	44	44	132	88	220	9	8	0	1	0	0	0.0	0.0
17	69	71	40	41	140	81	221	6	4	3	2	4	6	1.0	0.3

mean	**66.6**	**66.1**	**37.2**	**37.1**	**132.8**	**74.4**	**207.1**	**8.1**	**5.4**	**1.4**	**1.1**	**0.6**	**5.4**	**0.82**	**0.38**
± S.D.	6.7	9.0	9.9	10.6	14.6	19.9	22.3	4.7	2.7	1.6	1.0	1.4	4.9	0.54	0.20
Δ _norm _(%)	**-11%**	**-12%**	**-17%**	**-18%**	**-11%**	**-17%**	**-14%**	**19%**	**18%**	**10%**	**53%**	**2%**	**42%**		

MRC sum scores were used to describe muscle strength for right upper (RUE), left upper (LUE), right lower (RLE), left lower (LLE), upper (UE) and lower (LE) and all extremities (all E., see methods); disease related strength reduction is displayed as percent in the lower row. Sum scores of fasciculations, atrophy, bulbar signs, Babinski sign, spasticity and reflexes are represented as pathologic increase; the lower row values show percent of maximal pathologic scores. ALSFRS progression rates per month are calculated for the 6 months prior to MRI [PR/mth(6)] and the total disease duration [PR/mth(t)].

**Table 3:** Relative decrease in grey matter volumes in ALS patients compared to controls

Cluster	Side	Region	Brodmann's Area	MNI-Space			T-Value	P-Value
						
				*x*	*y*	*z*		
1	R	precentral gyrus	4	61	-6	20	7.29	0.011
	R	precentral gyrus	6	60	2	28	5.85	0.032
	R	precentral gyrus	6	60	-13	42	5.51	0.032
2	L	inferior parietal lobule	40	-51	-38	47	5.51	0.032
	L	postcentral gyrus	3	-62	-18	33	4.93	0.032
	L	postcentral gyrus	3	-58	-15	47	4.85	0.033
3	R	middle frontal gyrus	6	49	3	49	5.39	0.032
4	L	middle frontal gyrus	6	-46	0	43	5.09	0.032
5	R	postcentral gyrus	3	41	-25	46	5.03	0.032
6	R	postcentral gyrus	3	64	-23	41	5.01	0.032
	R	inferior parietal lobule	40	44	-58	51	4.48	0.039
7	R	superior frontal gyrus	8	36	19	53	4.99	0.032
8	L	postcentral gyrus	1	52	-23	54	4.83	0.033
9	L	middle frontal gyrus	9	42	33	30	4.64	0.035
	L	superior frontal gyrus	9	-36	36	38	4.21	0.048
10	R	superior frontal gyrus	8	9	34	57	4.55	0.037

Coordinates (in mm) of peak group differences in MNI space (Montreal Neurological Institute, ) are given for information. Significant local maxima more than 8 mm apart were translated to brain anatomy by converting their coordinates from MNI space to Talairach space  allowing for a search radius of 3 mm for nearest grey matter.
